# *FveARF2* negatively regulates fruit ripening and quality in strawberry

**DOI:** 10.3389/fpls.2022.1023739

**Published:** 2022-10-31

**Authors:** Shan-na Yi, Jian-xin Mao, Xin-yu Zhang, Xiao-ming Li, Zhi-hong Zhang, He Li

**Affiliations:** ^1^ Liaoning Key Laboratory of Strawberry Breeding and Cultivation, College of Horticulture, Shenyang Agricultural University, Shenyang, China; ^2^ Vegetable Research Institute, Liaoning Academy of Agricultural Sciences, Shenyang, China

**Keywords:** strawberry, FveARF2, fruit ripening, quality, potassium

## Abstract

Auxin response factors (ARFs) are transcription factors that play important roles in plants. ARF2 is a member of the ARF family and participates in many plant growth and developmental processes. However, the role of *ARF2* in strawberry fruit quality remains unclear. In this study, *FveARF2* was isolated from the woodland strawberry ‘Ruegen’ using reverse transcription-polymerase chain reaction (RT-PCR), which showed that *FveARF2* expression levels were higher in the stem than in other organs of the ‘Ruegen’ strawberry. Moreover, *FaARF2* was higher in the white fruit stage of cultivated strawberry fruit than in other stage. Subcellular localization analysis showed that FveARF2 is located in the nucleus, while transcriptional activation assays showed that FveARF2 inhibited transcription in yeast. Silencing *FveARF2* in cultivated strawberry fruit revealed earlier coloration and higher soluble solid, sugar, and anthocyanin content in the transgenic fruit than in the control fruit, overexpression of *FveARF2* in strawberry fruit delayed ripening and lower soluble solid, sugar, and anthocyanin content compared to the control fruit. Gene expression analysis indicated that the transcription levels of the fruit ripening genes *FaSUT1*, *FaOMT*, and *FaCHS* increased in *FveARF2-RNAi* fruit and decreased in *FveARF2-OE* fruit, when compared with the control. Furthermore, yeast one-hybrid (Y1H) and GUS activity experiments showed that FveARF2 can directly bind to the AuxRE (TGTCTC) element in the *FaSUT1*, *FaOMT*, and *FaCHS* promoters *in vitro* and *in vivo*. Potassium ion supplementation improved the quality of strawberry fruit, while silencing *FveARF2* increased potassium ion content in transgenic fruit. The Y1H and GUS activity experiments also confirmed that FveARF2 could directly bind to the promoter of *FveKT12*, a potassium transporter gene, and inhibited its expression. Taken together, we found that *FveARF2* can negatively regulate strawberry fruit ripening and quality, which provides new insight for further study of the molecular mechanism of strawberry fruit ripening.

## Introduction

Auxin response factors (ARFs) are typical transcription factors with a molecular weight of 67-129 KD (Remington et al., 2004). The ARF structure is highly conserved and consists of three domains: a DNA-binding domain (DBD), middle region (MR), and C-terminal dimerization domain (CTD) (Guilfoyle, 2015; Li et al., 2016). The DBD is an N-terminal-B3-like DNA binding domain that can bind to an auxin-response element (AuxRE: TGTCTC) (Tiwari et al., 2003). The MR region can be divided into the activation domain (AD) and repression domain (RD), according to the different amino acid sequences (Chandler, 2016). The CTD region is located at the C terminal end of the ARF protein and is responsible for protein-protein interactions (Li et al., 2021). To date, 23 *ARF* genes have been identified in *Arabidopsis thaliana*, along with *ARF5/6/7/8*, and *ARF19*, which is thought to be a transcriptional activator. The other ARFs are transcriptional suppressors based on the amino acid sequence in the MR region (Okushima et al., 2005b). In addition, 25 *ARF* genes in rice (*Oryza sativa*) (Wang et al., 2007), 21 *ARFs* in tomatoes (*Solanum lycopersicum*) (Wu et al., 2011), and 31 *ARFs* in apples (*Malus domestica*) (Luo et al., 2014) have been identified.

ARF is a key transcription factor involved in all stages of plant growth and development (Tian et al., 2004). *OsARF1* participates in rice coleoptile growth and in *Medicago truncatula*, *MtARF2/3/4* can regulate lateral root formation, while silencing *MtARF2/3/4* leads to shorter lateral roots (Waller et al., 2002; Kirolinko et al., 2021). In tomato, *SlARF5* can affect ovary development through gibberellin and auxin signaling pathways, and silencing *SlARF5* results in fruit size and weight reduction (Liu et al., 2018). Moreover, *SlARF7* also regulates fruit development through the gibberellin and auxin signaling pathways, and silencing *SlARF7* can reduce the cell division rate in tomato fruit epidermal cells (De Jong et al., 2009; De Jong et al., 2011). *SlARF9* is highly expressed in regions with high cell division activity, such as the axillary and root meristems, indicating that it can inhibit cell division (Mignolli et al., 2019). Plants that overexpress *SlARF9* form fruits that are smaller than the wild-type, whereas silencing *SlARF9* in tomatoes produces larger fruits than wild type (De Jong et al., 2015). *ARF10* is targeted by miR160 and mainly expressed in the pericarp of mature tomatoes (Damodharan et al., 2016). Overexpression of a mutated *ARF10* that cannot be targeted by miR160 can lead to seedless fruit or fruit with seeds that are unable to germinate (Hendelman et al., 2012). Moreover, *ARF16*, *ARF17*, and *ARF18* are differentially expressed during the fruit development and ripening stages (Xu et al., 2013; Zhao et al., 2021; Bi et al., 2022).

ARF2 is a member of the ARF family and a pleiotropic developmental regulator (Breitel et al., 2016). Previous studies have revealed that *ARF2* is involved in many plant growth and developmental processes (Ellis et al., 2005). In *Arabidopsis*, the inhibition of seedling hypocotyl growth by exogenous NAA (1-naphthlcetic acid) was enhanced in *arf2* mutants, suggesting that *ARF2* inhibits auxin signaling (Lim et al., 2010). Crosstalk between hormones is important for regulating plant growth processes, and *AtARF2* can influence ABA (Abscisic Acid) sensitivity by negatively regulating *AtHB33*, the expression of which is reduced by ABA (Wang et al., 2011). Potassium (K^+^) also plays a crucial role in plant growth and development, and *AtARF2* modulates the expression of the K^+^ transporter gene *AtHAK5* (High Affinity K^+^ transporter 5) in *Arabidopsis* (Zhao et al., 2016). Additionally, *ARF2* is involved in fruit ripening; there are two homologous genes, *SlARF2A* and *SlARF2B*, in tomatoes, both of which contribute to fruit ripening (Hao, 2014). Silencing either *SlARF2A* or *SlARF2B* can lead to fruit development defects, while silencing these genes simultaneously causes more serious defects, suggesting that *SlARF2A* and *SlARF2B* are functionally redundant during the fruit ripening process (Hao, 2014). In *SlARF2A* and *SlARF2B-RNAi* transgenic lines, dark green spots appear on the peel at both the immature and green ripening stages of tomato fruit development, which then develop into yellow or orange spots until the fruit is fully ripe (Hao et al., 2015). In papaya, CpARF2 can directly bind to CpEIL1 and enhance its transcriptional activity, thereby enhancing the expression of downstream ripening-related genes, such as *CpACS1*, *CpACO1*, and *CpXTH12*, and promoting fruit ripening (Liu et al., 2015; Zhang et al., 2020).

Strawberry is a typical non-respiratory climacteric fruit with high economic and nutritive value (Giampieri et al., 2012; Zeist and de Resende, 2019). The development and maturity of strawberry fruit are closely regulated by many factors, among which crosstalk between plant hormones plays an important role (Chai et al., 2011; Symons et al., 2012). Previous studies have revealed that auxin and ABA rather than ethylene play essential roles in the regulation of strawberry fruit ripening (Vallarino et al., 2015; Chai and Shen, 2016). The FaCHLH/ABAR signaling regulatory pathway responds to ABA signaling molecules and thus, affects fruit ripening (Jia et al., 2011). However, auxin is a key participant in regulating the early development of strawberry fruit, indicated by its high levels in the early green ripening period that decrease rapidly as the fruit develops and ripens (Nanao et al., 2014). In the early stages of strawberry fruit development, higher auxin levels inhibit the expression of ripening-related genes, such as *FaExps* and *FaXyl1*, whose activity affect strawberry fruit ripening as auxin levels decrease (Liu et al., 2011). In addition, many transcription factors, such as FveSEP3 (Pi et al., 2021), FaSAUR (Kiyak and Uluisik, 2022), and FaRIF (Martín-Pizarro et al., 2021), also influence strawberry fruit development. Although many transcription factors have been studied in strawberry fruit development, the function of ARF2 remains unclear.

To clarify the function of *ARF2* in strawberry fruit ripening, we cloned the coding sequence (CDS) of *ARF2* from the diploid strawberry (‘Ruegen’) and named it *FveARF2*. The relative expression levels of *FveARF2* in different organs and fruit development stages were analyzed. We then constructed *the FveARF2* silencing vector *35S::FveARF2-RNAi* and an overexpression vector *35S::FveARF2-OE*, which were injected into the white stage to obtain transgenic fruits. Phenotypic differences among these lines were analyzed using an empty vector as a control. Target genes for FveARF2 were searched and identified using yeast-one-hybrid and GUS activity experiments to explain the biological function of FveARF2. Our research aims to establish a solid foundation for further study of the function and mechanism of *FveARF2* in strawberries.

## Result

### Cloning and phylogenetic analysis of *FveARF2*

**Figure 1:**
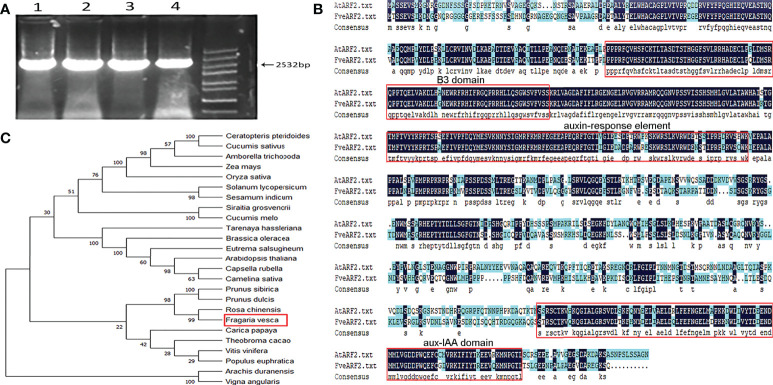
Amplification and structural and phylogenetic analysis of FveARF2. **(A)** Gene fragment amplification of the *FveARF2* CDS. Strips of 1-4 lines represent the *FveARF2* CDS region, which is 2532 bp in length. Marker: 5000 bp. **(B)** Structural and functional alignments of the FveARF2 and AtARF2 amino acid sequences. **(C)** Phylogenetic tree constructed from amino acid sequence alignments of ARF proteins.

Quality characteristics such as fruit sweetness and color are important factors in strawberry production and determine their economic value (Giampieri et al., 2012). Our previous study showed that potassium application increased the soluble sugar content of strawberry fruit (Zhou, 2019), and *ARF2* participates in potassium channels in *Arabidopsis* (Zhao et al., 2016). Therefore, the goal of this study was to determine whether *ARF2* can indirectly affect the fruit quality of strawberries and to investigate the function of the FveARF2 protein. We designed primers from the CDS of the *FveARF2* (XM_004297446.2) sequence provided in the NCBI (https://www.ncbi.nlm.nih.gov/) database and cloned *FveARF2* from the woodland strawberry ‘Ruegen’ by RT-PCR. The coding sequence of *FveARF2* was 2532 bp in length and encoded a protein of 843 amino acids (**Figure 1A**).

We used the DNAMAN software to conduct amino acid sequence analysis to further understand the conserved domains of FveARF2. The analysis revealed that FveARF2, another ARF2 family member, also contained a conserved B3, auxin-response element, and aux-IAA domains (**Figure 1B**). To further examine the evolutionary relationship between FveARF2 and its orthologs from multiple species, we performed multiple sequence alignment using full-length amino acid sequences. Using neighbor-joining analysis, we created a phylogenetic tree that indicated FveARF2 exhibited the closest genetic relationship with the *Rosa chinensis* auxin response factor 2 (XM_024309893.2) (**Figure 1C**). FveARF2 was also homologous to *Prunus dulcis* auxin response factor 2 (XP_034214955.1) and *Prunus sibirica* auxin response factor 2 (AVD68940.1), according to MEGA 6.0 (http://www.megasoftware.net/).

### Expression profiles of *FveARF2*

**Figure 2:**
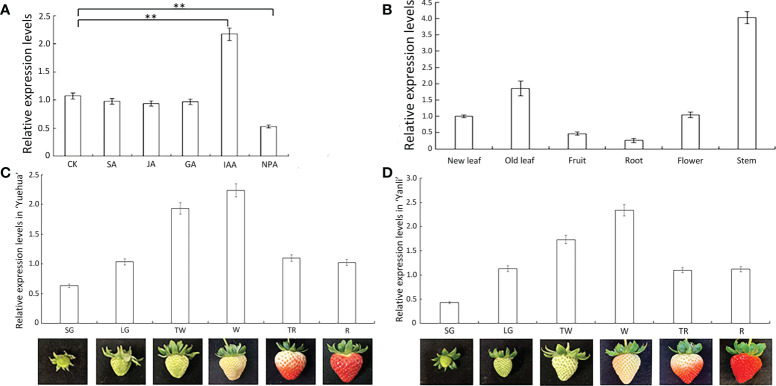
Relative expression characteristics of FveARF2. **(A)** Expression levels of *FveARF2* after different hormone treatments. **(B)** Expression levels of *FveARF2* in different organs of the ‘Ruegen’ cultivar. **(C, D)** Expression levels of *FveARF2* at different developmental stages of strawberry cultivars ‘Yuehua’ and ‘Yanli’ fruit. Values are the mean ± SD from three independent experiments with three biological replicates. ‘**’ Indicates very significantly different (P < 0.01, Duncan’s multiple range test).

To assess the importance of *FveARF2* in auxin response, we treated ‘Ruegen’ strawberry plants with 0.1 μmol·L^-1^ SA (salicylic acid), IAA (indoleacetic acid), JA (jasmonic acid), GA (gibberellic acid), and NPA (N-1-naphthylphthalamic acid) solutions, respectively, and used ‘Ruegen’ strawberry plants without exogenous hormone treatment as control. The qRT-PCR analysis of *FveARF2* expression showed that the relative expression level of *FveARF2* in IAA-treated strawberry plants was twice as high as that of the control plants, whereas NPA treatment inhibited the expression of *FveARF2* (**Figure 2A**).

We also analyzed the expression pattern of *FveARF2* in different organs and leaves at different development stages of the ‘Ruegen’ strawberry. We found that the expression level of *FveARF2* was lowest in the roots, while in stems and old leaves, *FveARF2* expression was 8- and 4-fold higher than that in roots, respectively (**Figure 2B**).

Strawberry fruit development can be divided into six stages: small green (SG), large green (LG), turning white (TW), white (W), turning red (TR), and red (R) (Wang et al., 2018). The relative expression levels of *FveARF2* in ‘Yuehua’ and ‘Yanli’ strawberry fruits at various developmental stages were analyzed using qRT-PCR. The results revealed that the relative expression level of *FveARF2* increased gradually in the early stages of fruit development and decreased in the late stages. Thus, *FveARF2* may contribute to the ripening process of strawberries fruits (**Figures 2C, D**).

### Subcellular localization and transcriptional activity of FveARF2

**Figure 3:**
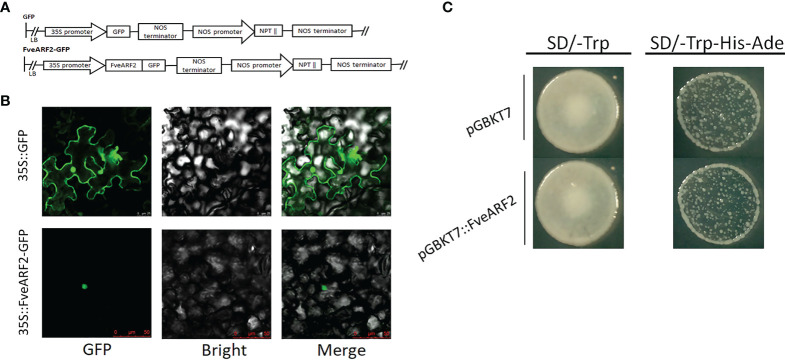
Subcellular localization and transcriptional activity of FveARF2. **(A)** Structural diagram of the 35S::GFP vector and the 35S::FveARF2-GFP construct. **(B)** Subcellular localization of the FveARF2 protein in *Nicotiana benthamiana* leaves. 35S::GFP (top) 35S::FveARF2-GFP were expressed in tobacco leaves (bottom), and visualized in the nucleus. Bar = 30 μm. **(C)** Yeast Y1H Gold cells transformed with pGBKT_7_::FveARF2 and analysis of these cells on SD/-Trp and SD/-Trp-His-Ade plates to evaluate whether FveARF2 has transcriptional activation activity.

As predicted by the website (http://www.cbs.dtu.dk/services/TargetP/), FveARF2 is located in the nucleus. To test this hypothesis, we expressed a FveARF2-green fluorescent protein (GFP) fusion construct driven by the constitutive CaMV 35S promoter (35S::FveARF2-GFP) in *Nicotiana benthamiana* leaves (**Figure 3A**). A CaMV 35S::GFP construct was used as a positive expression control. The fluorescent signal of 35S:: FveARF2-GFP was only detected in the nuclei of *N. benthamiana* leaf epidermal cells, while 35S::GFP alone was found throughout the entire cell, suggesting that FveARF2 specifically accumulated in the nucleus, which was consistent with expression patterns of other transcription factors (**Figure 3B**).

To study the transcriptional activity of FveARF2, we fused the CDS of *FveARF2* with GAL4 DBD to construct a pGBKT7-FveARF2 vector (pGBKT7::FveARF2). PGBKT7::FveARF2 and an empty vector (negative control) were transfected into the yeast system for transcriptional activity analysis. **Figure 3C** shows that yeast expressing pGBKT7::FveARF2 did not grow on SD/-Trp-His-Ade medium, but grew well on SD/-Trp medium. Yeast that expressed the pGBKT7 empty vector also only grew on SD/-Trp medium (**Figure 3C**). These results suggested that FveARF2 is not a transcriptional activator.

### FveARF2 affects strawberry fruit ripening and quality

**Figure 4:**
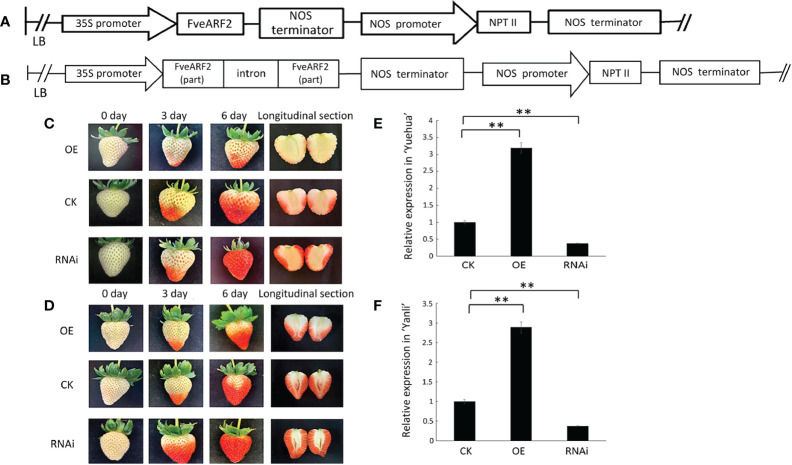
Phenotypic investigation of transgenic strawberry fruit. **(A)** Structural diagram of the vector used to overexpress *FveARF2* using the CaMV 35S promoter. **(B)** Structural diagram of the *FveARF2*-*RNAi* vector driven by the CaMV 35S promoter. **(C)** ‘Yuehua’ and **(D)** ‘Yanli’ fruit overexpressing *FveARF2* matured later, while silencing *FveARF2* induced fruit maturation in both cultivars when compared with the control. The relative expression of *FveARF2* in transgenic **(E)** ‘Yuehua’ and **(F)** ‘Yanli’ fruit, respectively. Values are the mean ± SD from three independent experiments with three biological replicates. ‘**’ Indicates very significant differences (P < 0.01, Duncan’s multiple range test).

We used Agrobacteria transient expression approach to analyze the biological functions of FveARF2. We created *FveARF2*-overexpression and *FveARF2-RNAi* plasmids (**Figures 4A, B**), which were infiltrated into octoploid ‘Yuehua’ and ‘Yanli’ strawberry fruit. A total of 70 berries from each cultivar was infiltrated, including 30 *FveARF2-OE* berries, 30 *FveARF2-RNAi* berries, and 10 empty vector controls (**Figures 4C, D**). The expression level of *FveARF2* was measured by qRT-PCR in the transgenic and control fruits, which was clearly higher in *FveARF2-OE* fruit and lower in *FveARF2*-RNAi fruit than in control fruit (**Figures 4E, F**). These results showed that *FveARF2*-RNAi berries ripened earlier than the control fruit, while *FveARF2-OE* fruit showed delayed ripening compared with the control. These results indicated that *FveARF2* can regulate fruit ripening in strawberries.

**Figure 5:**
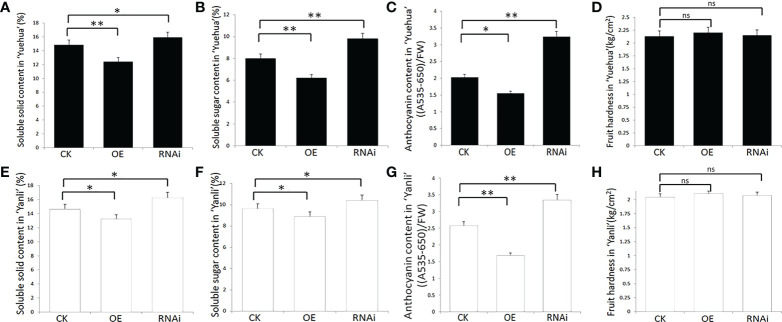
Physiological indexes of transgenic strawberry fruit in ‘Yuehua’ and ‘Yanli’. Soluble solid, soluble sugar, and anthocyanin content and fruit hardness in transgenic **(A-D)** ‘Yuehua’ and **(E-H)** ‘Yanli’ fruit, respectively. Values are the mean ± SD from three independent experiments with three biological replicates. ‘*’ Indicates significant difference (P < 0.05, Duncan’s multiple range test); ‘**’ indicates very significant differences (P < 0.01, Duncan’s multiple range test); ns indicates no significant difference.

To confirm this hypothesis, we measured the relative indices of strawberry fruit quality, including soluble solids, soluble sugars, anthocyanins, and fruit hardness. We found that these factors were higher in *FveARF2-RNAi* fruit than in *FveARF2-OE* fruit (**Figures 5A-H**). These experiments showed that *FveARF2* can negatively regulate strawberry fruit ripening.

**Figure 6:**
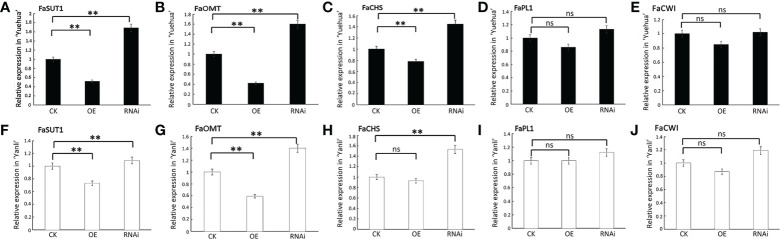
FveARF2 involved in strawberry fruit ripening by regulating fruit ripening related genes. Relative expression levels of *FaSUT1*, *FaOMT*, *FaCHS*, *FaPL1*, and *FaCWI* in *FveARF2*-OE and *FveARF2*-RNAi in **(A–E)** ‘Yuehua’ and **(F–J)** ‘Yanli’ fruits, respectively. Values are the mean ± SD from three independent experiments with three biological replicates. ‘**’ Indicates very significant differences (P < 0.01, Duncan’s multiple range test); ns indicates no significant difference.

Previous studies have shown that SUTs (Sucrose Transporters) are sucrose transporters responsible for transporting sucrose from phloem exosomes into the phloem (Borisjuk et al., 2002). Overexpression of *FaSUT1* in strawberry fruit may increase both sucrose and ABA content and accelerate fruit ripening (Jia et al., 2013). OMT (O-methyltransferase) is a flavonoid-modifying enzyme that determines the diversity of flavonoids (Borredá et al., 2022). The expression of *MdoOMT1* increases strongly with ethylene and is correlated with increased estragole production in ripened apples (Yauk et al., 2015). CHS (Chalcone Synthase) is the main synthase involved in anthocyanin synthesis. Comparative transcriptomics of wild and commercial citrus during early ripening showed that *CitCHS* is exclusively expressed in commercial varieties, in which it promotes the ripening process (Wang et al., 2018). PLs (Pectate Lyases) are a group of enzymes that cause pectate degradation in cell walls. Accumulation of the *Banl7* transcript, which encodes a protein homologous to pectate lyase, can be induced by the exogenous application of ethylene in green banana fruit (Dominguez-Puigjaner et al., 1997; Barros et al., 2016). CWI (Cell Wall Invertase) is the main protease involved in cell wall degradation during fruit overripening (Singh et al., 2010). To confirm that *FveARF2* regulates strawberry fruit ripening, we selected five ripening-related genes, *FaSUT1*, *FaOMT*, *FaCHS*, *FaPL1*, and *FaCWI*, and analyzed their relative expression levels in transgenic fruit. The qRT-PCR data indicated that the relative expression levels of *FaSUT1*, *FaOMT*, and *FaCHS* were lower in fruits overexpressing *FveARF2* when compared with the control, whereas they were clearly higher in *FveARF2-RNAi* fruit. During the experiment, *FaPL1* and *FaCWI* showed no significant changes in either *FveARF2-OE* or *FveARF2-RNAi* fruit compared to the control group (**Figure 6**). Therefore, we hypothesized that *FveARF2* plays a role in fruit ripening by influencing the expression of genes related to ripening.

**Figure 7:**
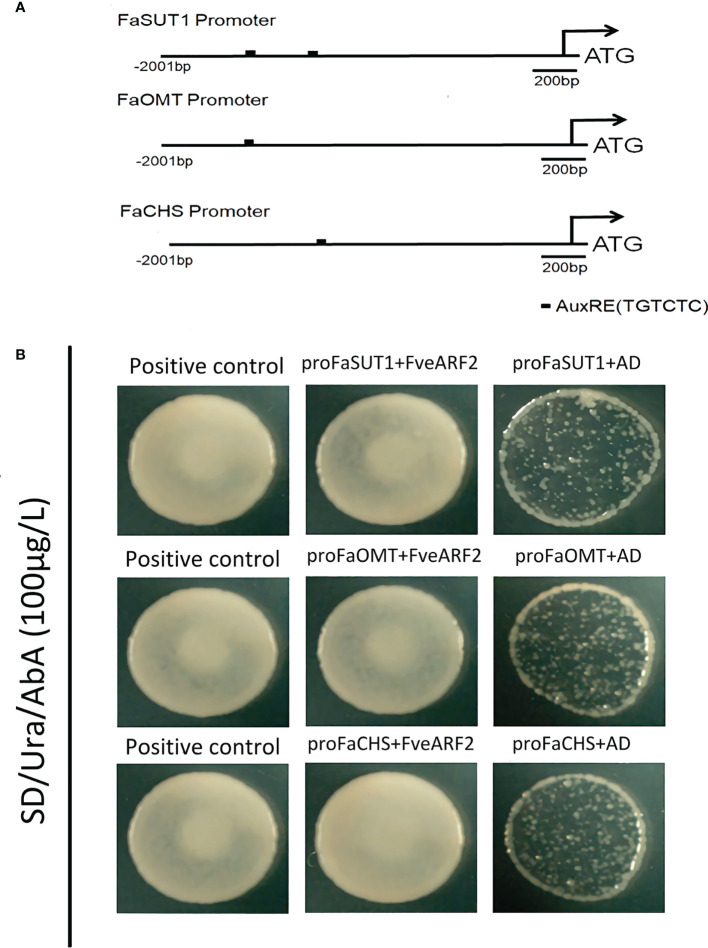
Y1H hybrid between FveARF2 and FaSUT1, FaOMT, and FaCHS promoters. **(A)** Structural diagram of *FaSUT1*, *FaOMT*, and *FaCHS* promoters. The block represents the AuxRE element (TGTCTC). **(B)** FveARF2 can bind to *FaSUT1*, *FaOMT1*, and *FaCHS* promoter *in vitro*. Positive control: p53::AbAi+p53::pGADT_7_.

**Figure 8:**
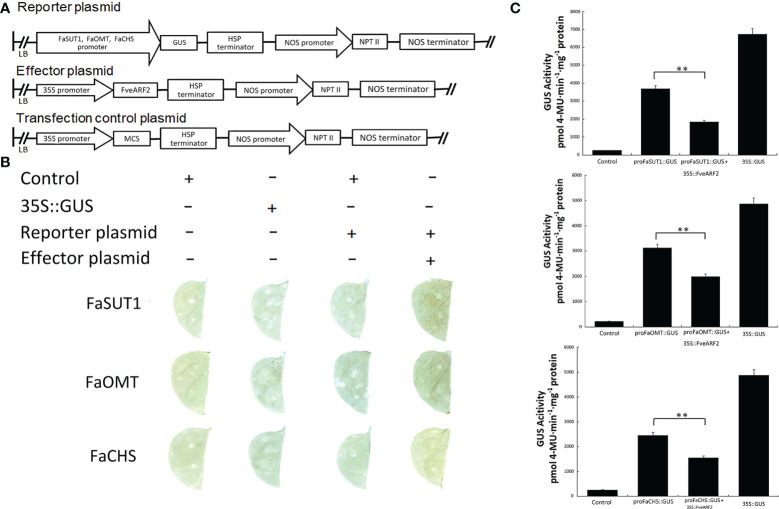
FveARF2 negatively regulates the expression of FaSUT1, FaOMT, and FaCHS by binding to their promoters. **(A)** Structural diagram of the reporter, effector, and transfection control plasmids. **(B, C)** GUS activity indicates an interaction between FveARF2 and the *FaSUT1*, *FaOMT*, and *FaCHS* promoters. The pRI201-AN and 35S::GUS vectors were used as negative and positive controls, respectively. *In vivo* GUS activity analysis showing that FveARF2 negatively regulates *FaSUT1*, *FaOMT*, and *FaCHS* expression. Values are the mean ± SD from three independent experiments with three biological replicates. ‘**’ Indicates very significant differences (P < 0.01, Duncan’s multiple range test).

Because FveARF2 is a transcription factor, we wanted to investigate whether FveARF2 could directly bind to the promoters of fruit ripening-related genes. We first analyzed the cis-acting elements in the promoters of *FaSUT1*, *FaOMT*, and *FaCHS*. We found that the promoters of *FaSUT1*, *FaOMT*, and *FaCHS* had AuxRE elements, indicating that FveARF2 may bind to the promoters of these genes (**Figure 7A**). To verify whether FveARF2 can bind to the *FaSUT1*, *FaOMT*, and *FaCHS* promoters, yeast one-hybrid (Y1H) tests and GUS activity analysis were performed. The Y1H assay showed that FveARF2 could directly bind to the AuxRE element in the *FaSUT1*, *FaOMT*, and *FaCHS* promoters in yeast (**Figure 7B**). *FaSUT1*, *FaOMT*, and *FaCHS* promoters were then individually inserted into the pRI201-GUS vector as reporters, and *FveARF2* driven by the 35S promoter and used as the effector in a transient tobacco leaf expression assay. We observed that FveARF2 inhibited the transcription of *FaSUT1*, *FaOMT*, and *FaCHS in vivo* (**Figures 8A–C**). The Y1H hybrid and GUS activity assays suggested that FveARF2 can directly bind to the *FaSUT1*, *FaOMT*, and *FaCHS* promoters and inhibit the transcription of their corresponding genes.

### FveARF2 also binds to the *FveKT12* promoter

**Figure 9:**
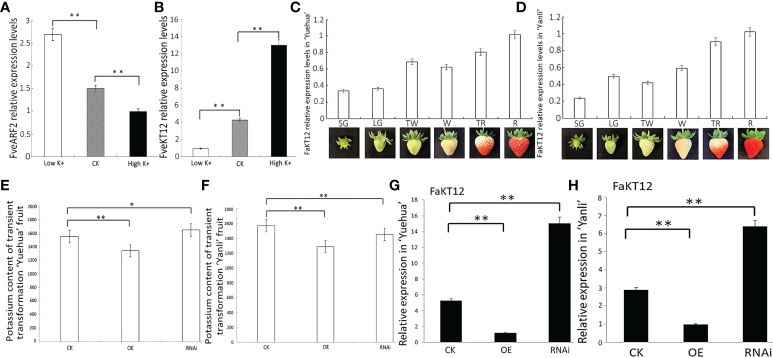
FveARF2 is involved in potassium transport in ‘Yuehua’ and ‘Yanli’ transgenic fruit. **(A)** *FveARF2* relative expression levels after 12 h of treatment with high K^+^ and low K^+^ conditions. **(B)** *FveKT12* relative expression levels after 12 h of high K^+^ and low K^+^ treatment. **(C, D)** *FaKT12* relative expression levels in different fruit development stages in ‘Yuehua’ and ‘Yanli’ strawberry cultivars. **(E, F)** Potassium content in transgenic ‘Yuehua’ and ‘Yanli’ fruit. **(G, H)** *FaKT12* relative expression levels in transgenic ‘Yuehua’ and ‘Yanli’ fruits. Values are the mean ± SD from three independent experiments with three biological replicates. ‘*’ Indicates significant differences (P < 0.05, Duncan’s multiple range test); ‘**’ indicates very significant differences (P < 0.01, Duncan’s multiple range test).

In *Arabidopsis*, AtARF2 regulates potassium ion (K^+^) transportation by directly binding to the promoter of the potassium transporter gene, *AtHAK5* (Wang and Wu, 2017). Potassium ions are important nutrients for strawberry fruit development. In our previous studies, we found that treatment with 8–10 mmol·L^-1^ K_2_SO_4_ had the greatest effect on improving soluble solids and soluble sugars in strawberry fruits (Zhou, 2019). To confirm whether FveARF2 can regulate the expression of potassium transporter genes, as well as AtARF2, we treated the diploid ‘Ruegen’ strawberry with high (40 mmol·L^-1^) and low K^+^ (10 mmol·L^-1^) supplemented MS media for 12 h, using a standard MS medium (about 20 mmol·L^-1^) as a control. The qRT-PCR results showed that high K^+^ treatment reduced the expression of *FveARF2* when compared with the control (**Figure 9A**). We downloaded the homologous sequence of *AtHAK5* in diploid strawberry from NCBI (https://www.ncbi.nlm.nih.gov/) and named this gene *FveKT12 (LOC101307201)*. Homology analysis showed 99.8% similarity in the nucleotide sequences of *FveKT12* and *FaKT12*. Amino acid sequence analysis indicated that the sequence consistency between AtHAK5 and FveKT12 was 44.71%; AtHAK5 and FveKT12 both have PLN00151 and PotE domains that participate in potassium transport. The expression levels of *FveKT12* under different K^+^ concentrations were analyzed by qRT-PCR to confirm that *FveKT12* is involved in potassium transport. The results revealed that the expression of *FveKT12* was higher in the high K^+^ treatment than in the low K^+^ treatment, indicating that *FveKT12* is involved in potassium transport in strawberry (**Figure 9B**).

In addition, qRT-PCR analysis of *FaKT12* expression at different fruit development stages showed that *FaKT12* expression increased during the fruit ripening process (**Figures 9C, D**), which was different from the expression profile of *FveARF2*. We analyzed the relative expression levels of *FaKT12* in transgenic fruits to confirm that FveARF2 affects fruit potassium content through the regulation of *FaKT12*. Lower expression of *FaKT12* was found in *FveARF2*-OE fruit (**Figures 9G, H**), and the potassium content in transgenic fruit was consistent with the gene expression results (**Figures 9E, F**). Together, these results confirmed that FveARF2 can reduce the transcription levels of *FaKT12* to negatively regulate potassium content in ‘Yuehua’ and ‘Yanli’ fruits.

**Figure 10:**
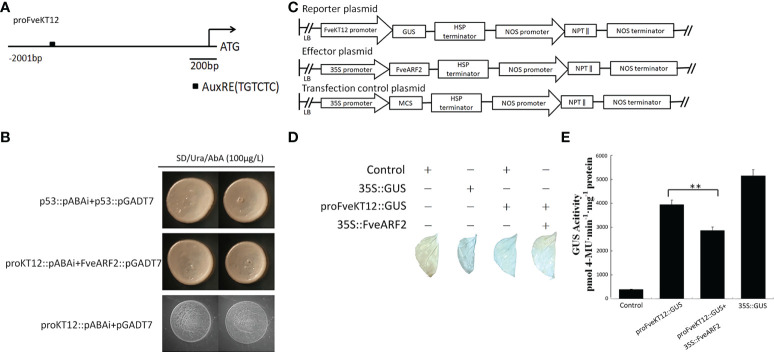
FveARF2 can bind to the FveKT12 promoter and negatively regulate its expression. **(A)** Structural diagram of the *FveKT2* promoter. The block represents the AuxRE element (TGTCTC). **(B)** FveARF2 can bind to *FveKT12* promoter *in vitro*. **(C)** Structural diagram of the reporter, effector, and control plasmids. **(D, E)** *In vivo* GUS activity analysis showing that FveARF2 negatively regulates *FveKT12* expression. Values are the mean ± SD from three independent experiments with three biological replicates. ‘**’ Indicates very significant differences (P < 0.01, Duncan’s multiple range test).

To verify whether FveARF2 can negatively regulate the expression of *FveKT12*, we downloaded and cloned the promoter of *FveKT12* from GDR (https://www.rosaceae.org/). Sequence analysis revealed that the *FveKT12* promoter contained an AuxRE element (**Figure 10A**), suggesting that FveARF2 could bind to the *FveKT12* promoter. To verify this hypothesis, yeast one-hybrid (Y1H) analysis and GUS activity analysis were performed. The Y1H assay indicated that FveARF2 could bind to the AuxRE element of the *FveKT12* promoter in yeast (**Figure 10B**). The *FveKT12* promoter was then individually inserted into the pRI201-GUS vector as a reporter, and *FveARF2* driven by the 35S promoter was used as the effector in a transient tobacco leaf expression assay. We observed that FveARF2 could also repress the transcription of *FveKT12 in vivo* (**Figures 10C-E**). The Y1H hybrid and GUS activity assays suggested that FveARF2 directly binds to the *FveKT12* promoter and inhibits its transcription.

## Discussion

### The expression characteristics of *FveARF2* in strawberry

ARFs are important response factors in the auxin signaling pathway and participate in the entire process of plant growth and development (Okushima et al., 2005a; Li et al., 2016). Previous studies have found that *AtARF2* is located on chromosome 5 in the *Arabidopsis thaliana* genome, and it was first characterized as a binding protein of ARF1 (Okushima et al., 2005a). Later studies confirmed that the *ARF2* gene is in at least 30 plant species, including tomato (*Solanum lycopersicum*) (Wu et al., 2011), (*Oryza sativa*) (Wang et al., 2007), apple (*Malus domestica*) (Luo et al., 2014), and grape (*Vitis vinifera*) (Wan et al., 2014).

In this study, we first amplified the CDS sequence of *FveARF2* from the ‘Ruegen’ strawberry (**Figure 1A**). The length of the coding region of *FveARF2* was 2532 bp, which encoded 843 amino acids. Similar to ARF2 in other species like *M. domestica* (Wang et al., 2022) and *A. thaliana* (Wang et al., 2011), FveARF2 also has the B3, auxin-response, and Aux-IAA domains (**Figure 1B**), and is localized to the nucleus (**Figure 3B**). In wheat, ARF2 has been reported to be an important transcription factor that is expressed in all tissues, but most highly expressed during floral development (Xu et al., 2020). In our study, we also found that *FveARF2* was expressed in all organ tissues, with the highest expression in stems and the lowest expression in roots of strawberry plants (**Figures 2B–D**). Additionally, the expression level of *FveARF2* in strawberry fruits gradually increases from the small green stage to the white stage of strawberry fruit development. However, *FveARF2* expression began to decrease and then stabilize when the fruit turned red (**Figures 2C, D**). This expression pattern indicated that *FveARF2* may play an important role in strawberry fruit ripening.

Plant hormones largely influence plant maturation, and previous studies have shown that *ARF2* is mainly involved in IAA and ABA pathways (Jong et al., 2011; Symons et al., 2012). In *Brassica napus*, both IAA and ABA treatments increased the expression level of *ARF2* (Xie et al., 2020). Moreover, silencing *ARF2* expression led to high sensitivity to exogenous ABA treatment in *Ginkgo biloba* (Chang et al., 2020). We found that IAA treatment strongly induced the relative expression of *FveARF2*, whereas NPA treatment inhibited its expression in strawberries. Therefore, we confirmed that FveARF2 is a transcription factor involved in the auxin-signaling pathway.

### FveARF2 negatively regulates fruit ripening and quality in strawberry

Fruit ripening is a complex physiological process for which many indicators are used to judge fruit maturity and quality, including peel color, hardness, and sugar content (Tiwari et al., 2003; Ayala-Zavala et al., 2004; Kang et al., 2013; Borredá et al., 2022). Studies have demonstrated the importance of *ARF2* in fruit ripening (Tian et al., 2004). For example, downregulation of *CpARF2* causes a significant increase in fruit hardness, which results in delayed ripening in papaya, while in durian, *DzARF2A* accelerates the ripening process by activating the auxin-ethylene crosstalk pathway (Zhang et al., 2020; Khaksar and Sirikantaramas, 2020).

Strawberries are important small berries that are cultivated worldwide (Giampieri et al., 2012). There are several current reports on strawberry fruit ripening, such as those that describe how ABA-mediated miR5290 promotes anthocyanin biosynthesis by inhibiting the expression of *FaMADS1* in postharvest strawberry fruit (Symons et al., 2012; Chen et al., 2022). The expression levels of *FaAux/IAA1* and *FaAux/IAA2* were also significantly downregulated during the ripening process in strawberry fruit, which was consistent with the trend of auxin content, further indicating that *FaAux/IAA1* and *FaAux/IAA2* regulate strawberry fruit development through the auxin response pathway (Liu et al., 2011). However, the function of *ARF2* in the ripening of strawberries has not been clarified. In this study, we found that *FveARF2* expression changed during the different developmental stages of strawberry fruit. To further clarify the biological function of *FveARF2* in strawberry fruit development, we constructed *FveARF2* overexpression and silencing constructs, which were used for a agrobacterium-mediated transient transformation experiment. Our findings suggest that overexpression of *FveARF2* can delay the redness of strawberries (**Figures 4C, D**), which corresponded to lower anthocyanin, soluble solids, and soluble sugar content in *FveARF2*-overexpression fruit than those in the control (**Figures 5A-C**, **E–G**). Conversely, silencing *FveARF2* promoted earlier fruit coloring (**Figures 4C, D**) and higher soluble solids, soluble sugar, and anthocyanin content in the transgenic fruit than in the control fruit (**Figures 5A–C**, **E–G**). These results indicate that *FveARF2* negatively regulates fruit ripening and quality in strawberries.

Several studies have identified a series of ripening-related genes that play significant roles in strawberry ripening (Chai et al., 2011). The expression of *FaSUT1* and *FaCWI* promote ripening by regulating sucrose metabolism, whereas *FaCHS* and *FaOMT* accelerate fruit development by increasing anthocyanin and flavonoid content. In addition, FaPL1 hydrolyzes pectin to expedite fruit softening (Hoffmann et al., 2006; Jia et al., 2016; Ibanez et al., 2019; Borredá et al., 2022). Therefore, to clarify the mechanism of *FveARF2* regulation of strawberry fruit ripening, we selected the *FaSUT1*, *FaCWI*, *FaCHS*, *FaOMT*, and *FaPL1* to determine whether *FveARF2* is directly involved in the regulation of fruit ripening. The expression levels of *FaSUT1*, *FaOMT*, and *FaCHS* were found to be lower in fruit overexpressing *FveARF2* than in the control, while they were upregulated in fruit that did not express *FveARF2* (**Figures 6A–C, F–H**). Additionally, Y1H and GUS activity analyses further confirmed that FveARF2 could directly bind to the promoters of *FaCHS*, *FaSUT1*, and *FaOMT1*, which inhibited their expression (**Figure 7B**, **8B, C**). Therefore, we concluded that *FveARF2* directly negatively regulates these ripening-related genes to inhibit strawberry fruit ripening. Similar to FveARF2, SlARF2A is a transcription inhibitor (Hao et al., 2015); however, unlike FveARF2, SlARF2A can promote the expression of key ethylene synthesis genes (e.g., *SlACO1* and *SlACS2/4*) and key fruit ripening regulators (e.g., *SlRIN*, *SlNOR*, and *SlCNR*) by negatively regulating the expression of an unknown transcription inhibitor to promote fruit ripening (Breitel et al., 2016). Additionally, CpARF2 can increase the accumulation of CpEIN1, which positively regulates the expression of key ethylene synthesis genes (e.g., *CpACS1* and *CpACO1*) by interfering with the ubiquitination and degradation of CpEIN1, which papaya fruit ripening (Zhang et al., 2020). SlARF2, CpARF2, and FveARF2 are transcriptional repressors but have different effects on fruit ripening. This may be because SlARF2 and CpARF2 regulate the expression of fruit ripening genes indirectly, whereas FveARF2 directly regulates fruit ripening genes. This conclusion will aid further exploration of the mechanism of *FveARF2* regulation of strawberry fruit ripening and quality.

### FveARF2 is also involved in potassium transport

Potassium is a quality element in fruit trees. It accounts for 2-10% of the dry weight in plants and its concentration in the cytoplasm is approximately 100 µmol·L^-1^ (Zhang et al., 2012). Potassium is involved in many physiological processes during plant growth and development, such as stomatal movement, membrane potential maintenance, stomatal reaction, cell elongation, and osmotic regulation (Ashley et al., 2006; Yang et al., 2009). In plants, K^+^ is transported through channel proteins and distributed by transport proteins (Maathuis and Sanders, 1997). Potassium transporters in plants include the four gene families: *KT/KUP/HAK*, *Trk/HKT*, *KEA*, and *CHX* (Mäser et al., 2001; Gierth and Mäser, 2007; Wang and Wu, 2013). Of these, the *KT/KUP/HAK* gene family has been widely studied in plants (Ashley et al., 2006). The *KT/KUP/HAK* gene family is composed of four gene clusters (I–IV) and includes 13 members in Arabidopsis and 27 members in rice (Rubio et al., 2000; Grabov, 2007; Amrutha et al., 2007; Gupta et al., 2008; Alemán et al., 2011). Previous studies have demonstrated that potassium ions are related to fruit quality and can affect fruit peel color and fruit sugar content (Lester et al., 2010).

In *Arabidopsis*, *AtHAK5* in the *KT/KUP/HAK* family can be regulated by AtARF2 (Zhao et al., 2016), thus affecting potassium ion transport. Our previous study revealed that soluble solids increased significantly in strawberries treated with potassium ions (Zhou, 2019). In this study, we found that potassium ion content decreased significantly in *FveARF2*-overexpression fruit, but increased significantly in fruit in which *FveARF2* expression was silenced (**Figures 9E, F**).

In strawberry, the CDS with the highest similarity to *AtHAK5* is *FveKT12/FaKT12*. Therefore, we analyzed the relative expression of *FaKT12* and found that it was lowest in the early stage of fruit development and gradually increased during the fruit ripening process (**Figures 9C, D**). The expression of *FaKT12* in *FveARF2*-overexpression fruit was significantly lower than that in the control, whereas it was significantly higher in *FveARF2*-silenced fruit than in the control (**Figures 9G, H**). Additionally, using yeast one-hybrid and GUS activity analyses indicated that FveARF2 can directly bind to the promoter of *FveKT12* and inhibit its expression (**Figures 10B–D**). According to *in silico* prediction and analysis, FveKT12 was located on the cell membrane. Therefore, we hypothesized that FveKT12 is localized on the cell membrane, where it is responsible for the transport of potassium ions, which affects the content of secondary metabolites in the fruit. However, the mechanism by which *FveKT12* participates in potassium ion transport remains unclear and requires further study.

**Figure 11:**
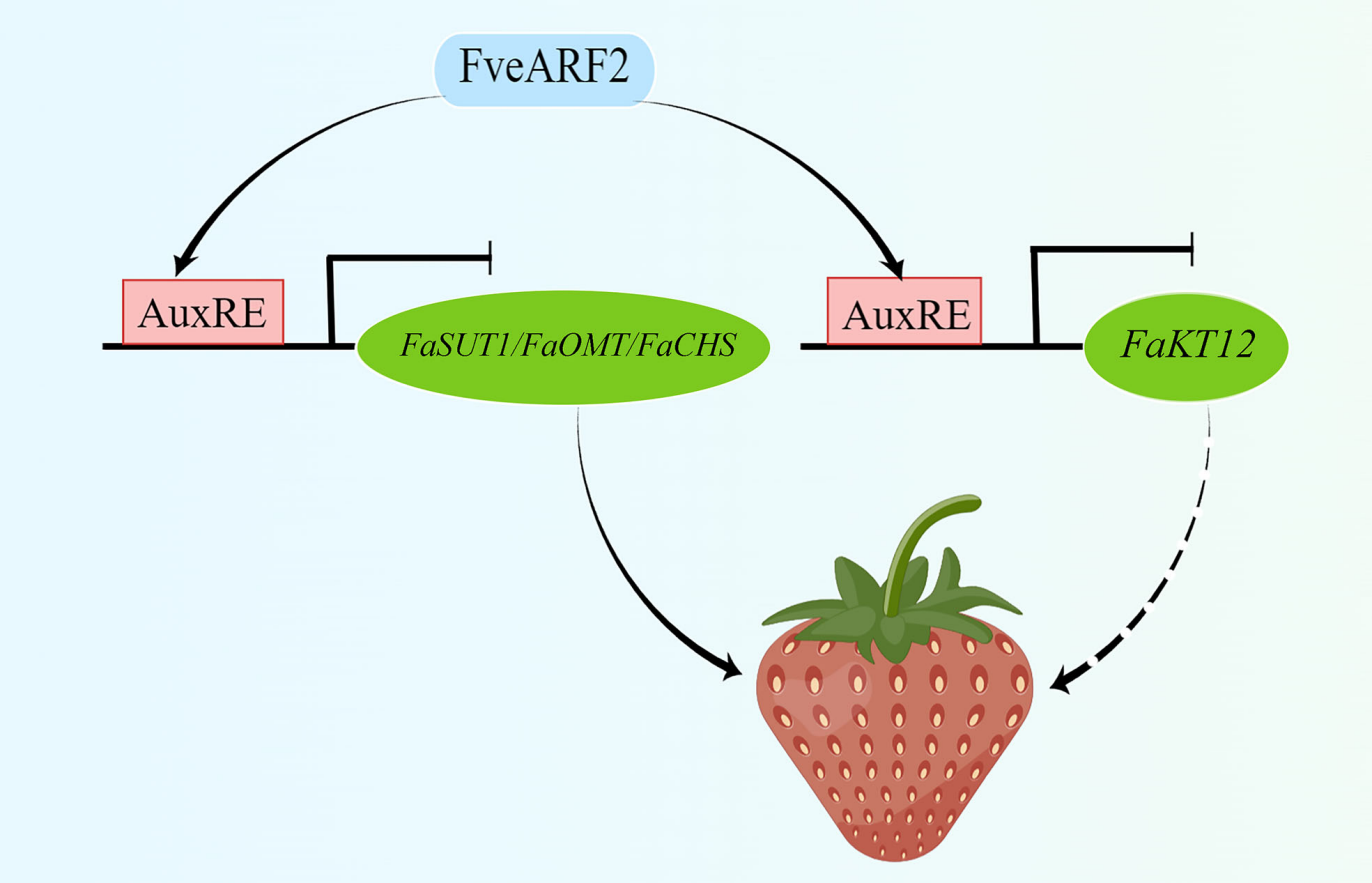
A model of FveARF2 as a negative regulator of fruit ripening and quality in strawberry. FveARF2 directly binds to the promoters of *FaCHS*, *FaSUT1*, and *FaOMT* and inhibits their expression; meanwhile, FveARF2 can also directly bind to the promoter of *FveKT12* and inhibit its expression.

In conclusion, we have proposed a model of *FveARF2* regulates fruit ripening and quality in strawberries (**Figure 11**). Our results show that FveARF2 directly binds to the promoters of *FaCHS*, *FaSUT1*, *FaOMT*, and *FveKT12* to inhibit their expression, which suggests these genes are targets for FveARF2-mediated regulation of fruit ripening and quality in strawberry.

## Materials and methods

### Plant materials and treatments

The ‘Ruegen’ woodland strawberry was grown in the solar greenhouse at the Shenyang Agriculture University (123°330 58″E, 41°490 9″N; Shenyang). New leaf (unexpanded green leaves, 30 days after sowing), old leaf (fully expanded green leaves, 105 days after sowing), fruit (red fruit, 105 days after sowing), root (main root, 105 days after sowing), flower (fully expanded flowers, 75 days after sowing) and stem (whole main stem, 105 days after sowing) were collected, frozen in liquid nitrogen, and stored at –80°C for gene cloning and expression pattern analysis.

Hormone treatment experiments used ‘Ruegen’ strawberries as the test material. We added 0.1 μmol·L^-1^ SA, JA, GA, IAA, and NPA were added to the liquid Murashige and Skoog (MS) medium (PH=5.8). The roots of ‘Ruegen’ strawberry plants were submerged into the medium and treated for 6 h. Leaves were collected, frozen in liquid nitrogen, and stored at –80°C for expression pattern analysis. The medium contained 18.8 mM KNO_3_; 20.6 mM NH_4_NO_3_; 1.3 mM KH_2_PO_4_; 1.5 mM MgSO_4_·7H_2_O; 3.0 mM CaCl_2_·2H_2_O; 0.005 mM KI; 0.1 mM MnSO_4_·4H_2_O, 0.1 mM CuSO_4_·5H_2_O, 0.1 mM H_3_BO_5_, 0.1 mM CoCl·6H_2_O, 0.03 mM ZnSO_4_·7H_2_O, 10 mM Na_2_MO_4_·2H_2_O, 0.1 mM FeSO_4_·7H_2_O, and 0.1 mM Na_2_EDTA. The potassium concentration of MS media conditions was increased to 40 mM for the high K^+^ treatment by adding KNO_3_, and the potassium concentration was decreased to 10 mM for the low K^+^ treatment by reducing KNO_3_ (Zhao et al., 2016). ‘Ruegen’ strawberries were treated for 12 h with high K^+^ (40 mM) or low K^+^ (10 mM), using normal MS medium (about 20 mM K^+^) as a control. Leaves were collected, frozen in liquid nitrogen, and stored at –80°C for expression pattern analysis.

Cultivated strawberries, ‘Yuehua’ and ‘Yanli’, were grown under greenhouse conditions at the Shenyang Agricultural University, China. The fruits were collected at six stages: small green (SG) (120 days after planting), large green (LG) (135 days after planting), turning white (TW) (145 days after planting), white (W) (152 days after planting), turning red (TR) (162 days after planting), and red (R) (170 days after planting), frozen in liquid nitrogen, and stored at –80°C for expression pattern analysis. Agrobacterium carrying the *FveARF2-OE* and *FveARF2-RNAi* plasmids was injected into fruits at the white and photos were taken 0, 3, and 6 d after injection. Each type of transgenic fruit was picked 6 d after injection and mixed, and then divided into three parts: part I (powdered samples stored at –80°C) for testing soluble solids, soluble sugar, and anthocyanin content, and fruit hardness; part II (powdered samples stored at –80°C) for testing gene relative expression levels; and part III (powdered samples stored at –80°C) for testing potassium content.

Seeds of *Nicotiana benthamiana* were sown on a peat:vermiculite:perlite = 3:3:1 substrate and were placed in a climate-controlled box under a long-day photoperiod at 24°C. Subcellular localization and GUS activity experiments were performed after approximately 1 mo of growth.

### Gene isolation

The CTAB (Cetyltrimethylammonium Bromide) method was used for total RNA (Ribonucleic acid) extraction, and the specific method used was the same as that used by Jordon-Thaden et al. (2015). Complementary DNA (cDNA) samples were obtained by reverse transcription using an RNA PCR kit (Takara, Dalian, China). The *FveARF2* CDS region was amplified by RT-PCR with the primers (FveARF2-F and FveARF2-R) (Tm: 65.48°C; Ta:57°C) that included *Kpn*I (GGTACC) and *Sal*I (GTCGAC) sites at the 5’ and 3’ ends (**Table S1**). PCR was performed according to the following procedure: (1) 95°C for 5 min, (2) 95°C for 30 s, 55°C for 30 s, and 72°C for 2 min 30 s with 35 cycles; and (3) 72°C for 10 min. The pMD18-T vector (Takara, Dalian, China) was used to load the *FveARF2* coding sequence, and the recombinant plasmid was transformed into *Escherichia coli* Trans5α competent cells. Positive colonies were selected after resistance screening with ampicillin and PCR validation, and then sequenced at BGI (Beijing Genomics institution)-Shenzhen. The amino acid sequence of FveARF2 was aligned with XP_004297494.1 from the *Fragaria vesca* Hawaii 4_1.0 genome, which was downloaded from NCBI (https://www.ncbi.nlm.nih.gov/) using DNAMAN 6.0 software (Lynnon Biosoft, USA) (Dong et al., 2021).

The CTAB method was used for total DNA (Deoxyribonucleic acid) extraction and the specific method as described by Porebski et al. (1997). The *FveKT12* promoter (2000 bp before transcription start site) was amplified by PCR using the primers (pFveKT12-F and pFveKT12-R) that included *Hind*III (AAGCTT) and *Xba*I (TCTAGA) sites at the 5’ and 3’ ends (**Table S1**). PCR was performed according to the following procedure: (1) 95°C for 5 min, (2) 95°C for 30 s, 55°C for 30 s, and 72°C for 2 min with 35 cycles; and (3) 72°C for 10 min. The pMD18-T vector was used for cloning and positive colonies were sequenced using the methods described above. The *FaCHS*, *FaOMT*, and *FaSUT1* promoters were also cloned by PCR using the above method and primers listed in **Table S1**.

### Phylogenetic analysis

The ARF2 amino acid sequences of *Zea mays (NM_001320084), Prunus sibirica (MF373591), Amborella trichopoda (XM_011629098), Oryza sativa (AB071293), Rosa chinensis (XM_024313067), Prunus dulcis (XM_034359064), Eutrema salsugineum (XM_006394382), Capsella rubella (XM_023777834), Carica papaya (XM_022055868), Arachis duranensis (XM_021129973), Sesamum indicum (XM_011084747), Solanum lycopersicum (DQ340255), Camelina sativa (XM_010459749), Vitis vinifera (XM_002284507), Tarenaya hassleriana (XM_010525193), Theobroma cacao (XM_018117665), Vigna angularis (XM_017583016), Cucumis melo (XM_008466144), Brassica oleracea* var. *oleracea (XM_013773223), Fragaria vesca subsp. Vesca (XM_004297446), Populus euphratica (XM_011041958), Cucumis sativus (AB112672), Siraitia grosvenorii (MK404723), Ceratopteris pteridoides (KX784815), Arabidopsis thaliana (NM_125593)* were obtained from the NCBI nucleotide database (http://www.ncbi.nlm.nih.gov/nucleotide/). MEGA version 6.0 was used for phylogenetic tree construction with the neighbor-joining (NJ) method (Tamura et al., 2013).

### Subcellular localization of FveARF2

Subcellular localization was performed using the Dong et al. (2021). Primers (FveARF2-GFP-F and FveARF2-GFP-R) were designed to amplify the *FveARF2* CDS region without a termination codon (**Table S1**). The LA Taq polymerase (Takara, Dalian, China) was used for PCR, which was performed according to the following procedure: (1) 95 C for 5 min, (2) 95°C for 30 s, 55°C for 30 s, and 72°C for 2 min 30 s for 35 cycles, and (3) 72°C for 10 min. *Xba*I and *BamH*I were used to digest the pRI101-GFP vector and *35S::FveARF2-GFP* (FveARF2-GFP) was constructed using T_4_ DNA ligase (Takara, Dalian, China). The pRI101-GFP vector containing 35S::GFP (GFP) was used as a control. FveARF2-GFP and pRI101-GFP were transformed into the Agrobacterium strain GV3101, and the bacterial liquid culture was used to inoculate *N. benthamiana* leaves. A laser confocal fluorescence microscope (TCS SP8-SE; Leica, Wechsler, Germany) was used to examine GFP fluorescence at an excitation wavelength of 488 nm and an emission wavelength of 505–530 nm.

### Transcriptional activation analysis of FveARF2

The transcriptional activity of *FveARF2* was determined by observing yeast growth as described by Zhang et al. (2019). The *FveARF2* CDS was inserted into the pGBKT_7_ vector to create *pGBKT_7_::FveARF2* (pGBKT_7_-FveARF2) constructs with the following primers: FveARF2-BD-F/R (**Table S1**). The pGBKT_7_-FveARF2 and pGBKT_7_ constructs were then transformed into Y1H Gold yeast competent cells using a yeast transformation kit (Shaanxi Protein Interaction Biotechnology Company Limited, Shaanxi, China). SD/-Trp medium was used to select the transformed yeast strains, and SD/-Trp-His-Ade medium was used to examine transcriptional activity.

### Quantitative RT-PCR analysis

Using SYBR Green II (Takara, Dalian, China) as a fluorescent dye, qRT-PCR was performed using a 7500 system (Applied Biosystems, Foster City, CA, USA). The qRT-PCR primers used for *FveARF2*, *FveKT12*, *FaKT12*, *FaCHS*, *FaOMT*, *FaSUT1, FaPL1*, and *FaCWI* are listed in **Table S1** (FveARF2-QF/QR, FveKT12-QF/QR, FaKT12-QF/QR, FaCHS-QF/QR, FaOMT-QF/QR, FaSUT1-QF/QR, FaPL1-QF/QR, FaCWI-QF/QR). The experiments were conducted using three biological replicates, and the results were normalized using 26S rRNA as the housekeeping gene (Li et al., 2017). The qRT-PCR analysis of *Fve26s* or *Fa26s* was conducted using the following primers: Fve26s-QF/QR or Fa26s-QF/QR (**Table S1**). PCR was performed using the following thermal cycling conditions: 50°C for 2 min, 95°C for 10 min, followed by 40 cycles of 95°C for 15 s, and 60°C for 1 min. The transcription levels were calculated using the 2^–ΔΔCT^ method (ΔΔCT=ΔCT (test) - ΔCT (calibrator); ΔCT (test) =CT (targer, test) -CT (ref, test); ΔCT (calibrator) =CT (targer, calibrator) -CT (ref, calibrator)) (Chang et al., 2007).

### Transient transformation of strawberry fruit

The *FveARF2* CDS region with added *Kpn*I and *Sal*I restriction sites was inserted into the pRI101-AN vector to form the 35S::*FveARF2* (FveARF2-OE) overexpression vector. The *FveARF2*-RNAi vector was constructed by inserting a 300 bp *FveARF2* gene into the left (*Xba* I and *Sal* I) and right (*Kpn* I and *Sac* I) multiple cloning sites of the pART27 vector. *FveARF2-OE*, *FveARF2-RNAi*, and empty vectors were introduced into Agrobacteria strain GV3101 using the freeze-thaw method. Agrobacteria cells were cultured to an optical density of 0.8 at 600 nm (OD_600_) and Agrobacteria infection was performed as described by Zhang et al. (2019). Strawberries were treated by whole fruit injection with 1 mL of Agrobacteria suspension.

### Determination of related indexes of fruit quality

The content of soluble solids in strawberry juice was detected by pocket digital Abbe refraction (ATAGO, Japan) at ambient temperature.

The hardness of the strawberries was measured using an RT hardness tester. The ratio of the pressure per unit area to that per unit area was defined as the fruit hardness. We calculated fruit hardness = n/s, where n is the force of the fruit force spring and s is the force area of the fruit during measurement. The unit of the calculation result was kg/cm^2^.

Soluble sugar content in strawberry fruit was determined using Anthrone colorimetry and the anthocyanin content in fruit was determined by colorimetry. The anthocyanin in fruit samples was extracted using an extraction solution (1% hydrochloric acid, 18% propanol, 81% ddH_2_O), and the absorbance values at A535 nm and A650 nm were measured using a UV spectrophotometer, as previously described by Chai et al. (2011).

### Yeast one-hybrid assay

The *FveARF2* sequence was ligated into the pGADT_7_ vector to create pGADT_7_-FveARF2, using the following primers: FveARF2-AD-F/R (**Table S1**). The promoter sequences (2000 base pairs before transcription start site) of *FveKT12*, *FaOMT*, *FaSUT1*, and *FaCHS* were cloned and analyzed to determine the presence of AuxREs. The *FveKT12*, *FaOMT*, *FaSUT1*, and *FaCHS* promoter fragments were then amplified and individually inserted into the pAbAi vector to obtain pAbAi-FveKT12, pAbAi-FaOMT, pAbAi-FaSUT1, and pAbAi-FaCHS using the primer pairs pFveKT12-A-F/R, pFaOMT-A-F/R, pFaSUT1-A-F/R, and pFaCHS-A-F/R (**Table S1**), respectively. The vectors containing *FveARF2* and fragments of the *FveKT12*, *FaOMT*, *FaSUT1*, or *FaCHS* promoters were co-transformed into the Y1H yeast strain, and the Y1H assay was performed as previously described by Li et al. (2018).

### GUS staining and activity detection

GUS staining and activity assays were performed as described by Jefferson et al. (1987). The *FveARF2* sequence was ligated into the pRI101-AN vector, while the *FveKT12*, *FaOMT*, *FaSUT1*, and *FaCHS* promoter fragments were amplified and individually inserted into the pRI201-GUS vector using the primer pairs pFveKT12-F/R, pFaOMT-F/R, pFaSUT1-F/R, and pFaCHS-F/R (**Table S1**), respectively. Using pRI201-GUS containing 35S::GUS as a control, the vectors were infiltrated into *N. benthamiana* leaves. After three days, leaves were treated with GUS staining solution (100 mM sodium phosphate buffer, 0.1% Triton X-100, 0.1% N-laurylsarcosine, 10 mM Na_2_EDTA, 1 mM K_3_Fe(CN)_6_, 1 mM K_4_Fe(CN)_6_, 0.5 mg·mL^-1^ X-Gluc) for 24 h in the dark. Leaves were decolorized with 70% alcohol until the control sample was transparent. Total protein was extracted from the leaves, and GUS protein content was determined as described by Bradford (1976). Protein concentration and enzyme activity were measured using a Cary Eclipse fluorescence spectrophotometer.

### Statistical analysis

The significance of the differences between the means was analyzed using Duncan’s test in the DPS 7.05 software (Dong et al., 2022). Mean values marked with * and ** indicate significant differences at the 5% and 1% levels, respectively.

## Data availability statement

The datasets presented in this study can be found in online repositories. The names of the repository/repositories and accession number(s) can be found in the article/**Supplementary Material**.

## Author contributions

HL and ZZ designed this project. SY and JM performed the experiments. HL, XZ, and XL analyzed the data. HL, SY, and JM wrote the manuscript. All authors contributed to the article and approved the submitted version.

## Funding

This work was supported by the National Key R&D Program of China (2019YFD1000200); the National Natural Science Foundation of China (31872069, 32130092); the Liaoning Key R&D Program (2020JH2/10200032); the Shenyang Young and Middle-aged Science and Technology Innovation Talents Support Plan (RC190446); and the Liaoning BaiQianWan Talents Program (2016921067).

## Conflict of interest

The authors declare that the research was conducted in the absence of any commercial or financial relationships that could be construed as a potential conflict of interest.

## Publisher’s note

All claims expressed in this article are solely those of the authors and do not necessarily represent those of their affiliated organizations, or those of the publisher, the editors and the reviewers. Any product that may be evaluated in this article, or claim that may be made by its manufacturer, is not guaranteed or endorsed by the publisher.
