# Supplementary material for: FveARF2 negatively regulates fruit ripening and quality in strawberry
Source: Front Plant Sci. 2022 Oct 31;13:1023739. doi: 10.3389/fpls.2022.1023739 (PMC9660248; doi:10.3389/fpls.2022.1023739)
Supplement: Supplementary file 1 [file Table_1.docx]

**Table S1** Primers used in this study

| Primers | Sequence（5’→3’） | Use |
| --- | --- | --- |
| FveARF2-F | GGGGTACCATGACGTCATCGGAGGTT | Clone of *FveARF2* CDS/  Construction of *FveARF2*-OE vector |
| FveARF2-R | CGAGCTCTCACTGAGACTTCCCCTCT |  |
| FveARF2-GFP-F | GCTCTAGAATGACGTCATCGGAGGTT | Construction of *FveARF2*-GFP vector |
| FveARF2-GFP-R | CGGGATCCCTGAGACTTCCCCTCTCTT |  |
| pRNAi-FveARF2-F1 | GCTCTAGAACCACAACGACGGCAGGAAT | Construction of *FveARF2*-RNAi vector |
| pRNAi-FveARF2-R1 | TCCCCCGGGGCGCAAACACCTCGTCTGTATC |  |
| pRNAi-FveARF2-F2 | GCTCTAGAACCACAACGACGGCAGGAAT |  |
| pRNAi-FveARF2-R2 | CGAGCTCACCACAACGACGGCAGGAAT |  |
| FveARF2-BD-F | GGAGGCCGAATTCCCATGACGTCATCGGAGGTTTC | Construction of pGBKT_7_-FveARF2 vector |
| FveARF2-BD-R | GGTCGACGGATCCCCTCACTGAGACTTCCCCTCTC |  |
| FveARF2-AD-F | CGGGATCCATGACGTCATCGGAGGTT | Construction of pGADT_7_-FveARF2 vector |
| FveARF2-AD-R | CCCTCGAGCAAGGTCACTGAGACTTC |  |
| FveARF2-QF/ FaARF2-QF | TCATGTCAAGCTACCCGTGA | qRT-PCR of *FveARF2/ FaARF2* |
| FveARF2-QR/FaARF2-QR | AATAACCGTCTCCGTCTCCC |  |
| Fve26S-QF/ Fa26S-QF | TAACCGCATCAGGTCTCCAA | qRT-PCR of *Fve26S/ Fa26S* |
| Fve26S-QR/ Fa26S-QR | CTCGAGCAGTTCTCCGACAG |  |
| FveKT12-QF/FaKT12-QF | GTACTACTTCATCTCAATC | qRT-PCR of *FveKT12/ FaKT12* |
| FveKT12-QR/ FaKT12-QR | CGTCTCGATAGCATATGTAG |  |
| FaSUT1-QF | CTTAGGTACAACTTAGGCAT | qRT-PCR of *FaSUT1* |
| FaSUT1-QR | CATTCATATTGGCAGAGCCA |  |
| FaOMT-QF | TGCATGGTCACCATTGGTAG | qRT-PCR of *FaOMT* |
| FaOMT-QR | GTACGTGCACCGTGATGGA |  |
| FaCHS-QF | TTGCACCGAATAGGTACTC | qRT-PCR of *FaCHS* |
| FaCHS-QR | CATGTGATTCGACGGAGTTA |  |
| FaPL1-QF | CGATTGTACCATTAGATCATC | qRT-PCR of *FaPL1* |
| FaPL1-QR | CTGTACAGTCAGTCAATTGAT |  |
| FaCWI-QF | TGAGACTAGTCAATTCCATGT | qRT-PCR of *FaCWI* |
| FaCWI-QR | AGGATCAGTAACATGGATCA |  |
| pFveKT12-F | CCAAGCTTGCTGTGAGTGGTCTGCAGGG | Clone of *FveKT12* promoter/ Construction of proFveKT12-GUS vector |
| pFveKT12-R | GCTCTAGAAATATGTAGGAAGGAGGAGG |  |
| pFveKT12-A-F | CCAAGCTTTCCTGAGTGTAACATGCT | Construction of proFveKT12-AbAi vector |
| pFveKT12-A-R | CCCTCGAGCCTGCATGAAGTATATAA |  |
| pCHS-F | CCAAGCTTACAATCAATTTGCTAATATC | Clone of *FaCHS* promoter/ Construction of proFaCHS-GUS vector |
| pCHS-R | GCGGATCCTTATTTGAAAGTTATTAAAT |  |
| pCHS-A-F | CCAAGCTTACAATCAATTTGCTAATATC | Construction of proFaCHS-AbAi vector |
| pCHS-A-R | CCCTCGAGTTATTTGAAAGTTATTAAAT |  |
| pOMT-F | CCAAGCTTTGTTTGTTATTGTGAATGCG | Clone of *FaOMT* promoter/ Construction of proFaOMT-GUS vector |
| pOMT-R | GCGGATCCGGTTATTATGAGCCCAGATG |  |
| pOMT-A-F | CCAAGCTTTGTTTGTTATTGTGAATGCG | Construction of proFaOMT-AbAi vector |
| pOMT-A-R | CCCTCGAGGGTTATTATGAGCCCAGATG |  |
| pSUT1-F | CCAAGCTTAAGGGCGACATAAAGTAGTT | Clone of *FaSUT1* promoter/ Construction of proFaSUT1-GUS vector |
| pSUT1-R | GCGGATCCCTACTTGAACTGACTTGACA |  |
| pSUT1-A-F | CCAAGCTTAAGGGCGACATAAAGTAGTT | Construction of proFaSUT1-AbAi vector |
| pSUT1-A-R | CCCTCGAGCTACTTGAACTGACTTGACA |  |
